# Supplementary figures and images for: An ISR-independent role of GCN2 prevents excessive ribosome biogenesis and mRNA translation
Source: Life Sci Alliance. 2025 Mar 3;8(5):e202403014. doi: 10.26508/lsa.202403014 (PMC11876863; doi:10.26508/lsa.202403014)

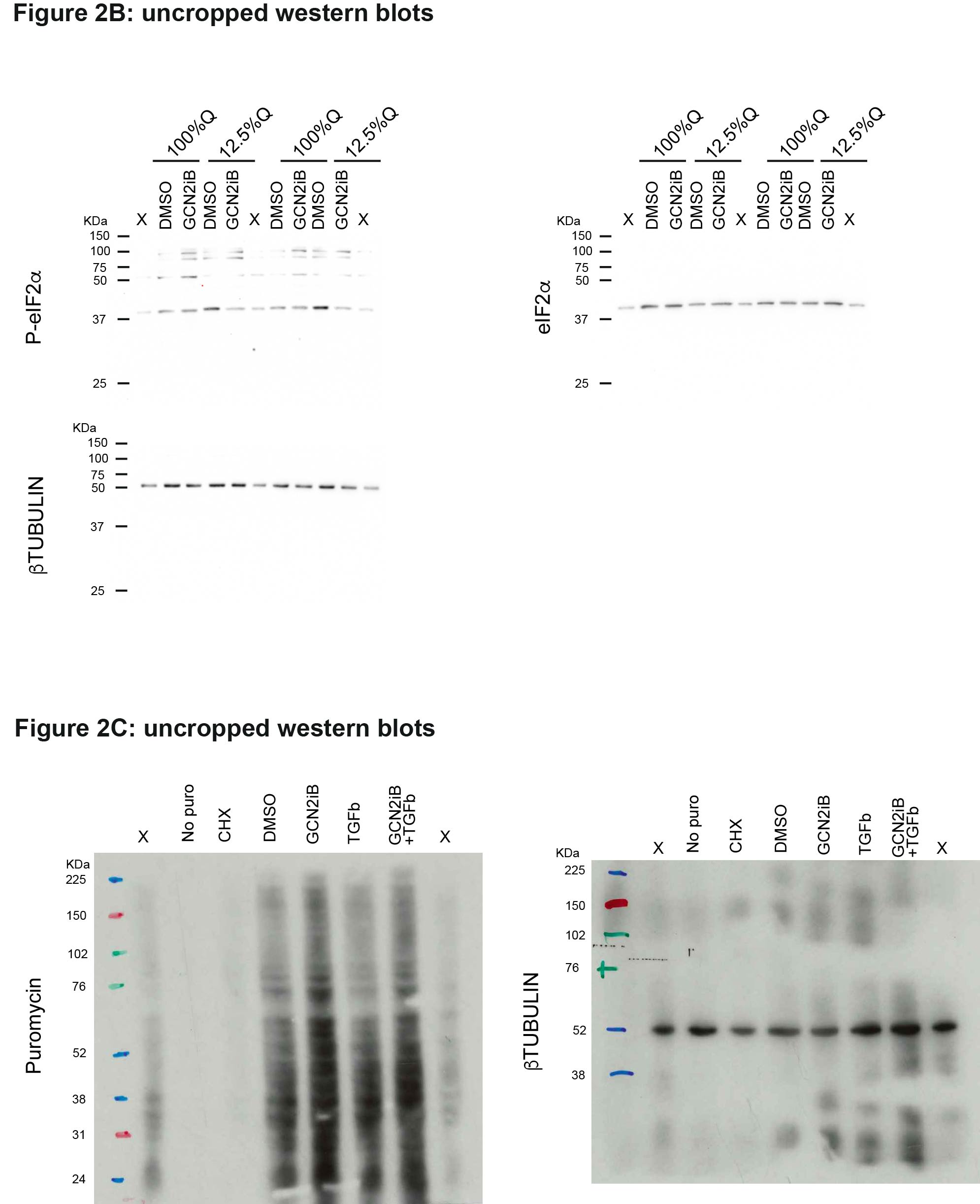

Supplement: Supplementary file 1 [file LSA-2024-03014_SdataF2.1.jpg]

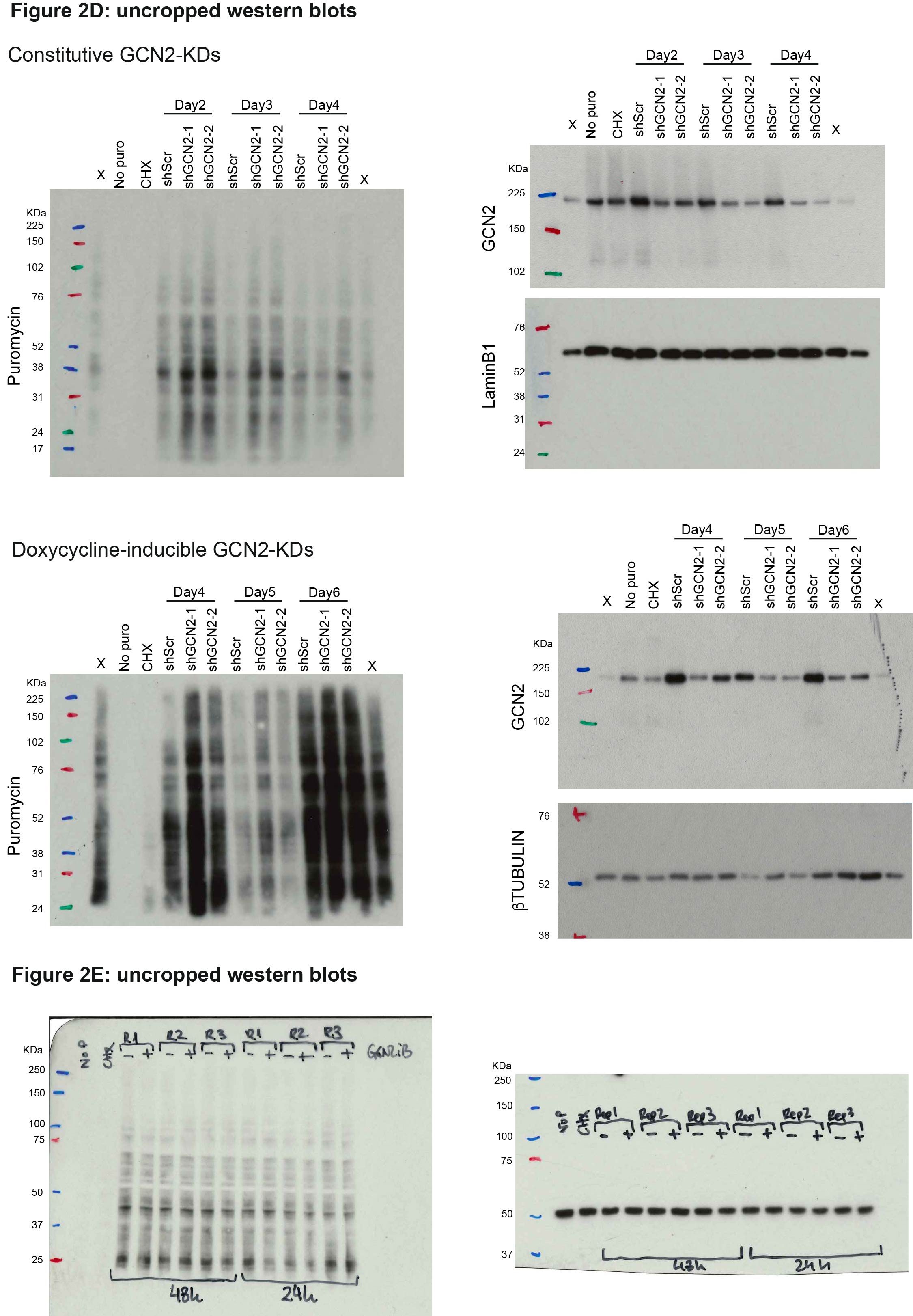

Supplement: Supplementary file 2 [file LSA-2024-03014_SdataF2.2.jpg]
